# Mucus threads from surface goblet cells clear particles from the airways

**DOI:** 10.1186/s12931-021-01898-3

**Published:** 2021-11-25

**Authors:** Anna Ermund, Lauren N. Meiss, Brendan Dolan, Florian Jaudas, Lars Ewaldsson, Andrea Bähr, Nikolai Klymiuk, Gunnar C. Hansson

**Affiliations:** 1 Department of Medical Biochemistry and Cell Biology, University of Gothenburg, PO Box 440, 405 30 Gothenburg, Sweden; 2 Institute of Molecular Animal Breeding and Biotechnology, Gene Center, Ludwig-Maximilians-University Munich, Munich, Germany; 3 Experimental Biomedicine, University of Gothenburg, Gothenburg, Sweden

**Keywords:** Mucins, Respiratory tract, Mucus bundle, Goblet cells, Trachea, Bronchi

## Abstract

**Background:**

The mucociliary clearance system driven by beating cilia protects the airways from inhaled microbes and particles. Large particles are cleared by mucus bundles made in submucosal glands by parallel linear polymers of the MUC5B mucins. However, the structural organization and function of the mucus generated in surface goblet cells are poorly understood.

**Methods:**

The origin and characteristics of different mucus structures were studied on live tissue explants from newborn wild-type (WT), cystic fibrosis transmembrane conductance regulator (CFTR) deficient (CF) piglets and weaned pig airways using video microscopy, Airyscan imaging and electron microscopy. Bronchoscopy was performed in juvenile pigs in vivo.

**Results:**

We have identified a distinct mucus formation secreted from the surface goblet cells with a diameter less than two micrometer. This type of mucus was named mucus threads. With time mucus threads gathered into larger mucus assemblies, efficiently collecting particles. The previously observed Alcian blue stained mucus bundles were around 10 times thicker than the threads. Together the mucus bundles, mucus assemblies and mucus threads cleared the pig trachea from particles.

**Conclusions:**

These results demonstrate that normal airway mucus is more complex and has a more variable structural organization and function than was previously understood. These observations emphasize the importance of studying young objects to understand the function of a non-compromised lung.

**Supplementary Information:**

The online version contains supplementary material available at 10.1186/s12931-021-01898-3.

## Background

The mucosal surfaces of the airways and lungs are exposed to the environment with airborne pollutants, particles and microbes. The mucociliary system protects the lungs from these inhaled particles and microbes and the coordinated beating of multiple cilia drives the flow of liquid cephalad. However, the normal mucus components are more complex and diverse than normally appreciated. The submucosal glands of humans and pigs produce mucus bundles made from linear polymers of the MUC5B mucin [1]. These bundles are important for airway clearance as they clear large particles (0.5–0.6 mm) and bacteria [2, 3] and genetic deletion of submucosal glands in pigs further emphasize their importance for removal of bacteria and particles [4].

Normal mucus is transparent due to high water content and it is thus challenging to study the mucus in its native form and in its natural environment. Using the cationic dye Alcian blue to stain mucus bundles, we quantified the thickness and velocity of the bundles and demonstrated that they were two to three times thicker than the airway surface liquid (ASL). This conclusion is based on our published values for Alcian blue positive mucus bundle thickness, approximately 27 µm [3] and ASL thicknesses measured using µOCT [5, 6]. The Alcian blue positive mucus bundles move intermittently and perpendicular to the liquid flow, suggesting that they move by an attachment/detachment mechanism and are intermittently attached to the surface. We proposed that this attachment was mediated by the MUC5AC mucin extending from the surface goblet cells and coating the bundles [7]. The mucus bundles were immobile in newborn CF piglets [3] and also retained at the gland openings [8].

We have previously demonstrated that submucosal gland-made mucus bundles moved slower than 40 nm carboxylated fluorescent beads [3]. Furthermore, the mucus bundle movement was arrested upon stimulation with the acetylcholine analog carbachol, whereas the velocity of the fluorescent beads increased on both WT and CF newborn piglet tracheas [3]. The beads were collected on shapes that we previously called “bead-collecting strands” [3]. To better describe their morphology and keep the nomenclature used by others [8, 9], we will use the name mucus threads. We hypothesized that as these components were able to collect the negatively charged beads, they may have a physiological function in the clearance system of the lungs. To test this hypothesis, we have now investigated the origin of the mucus threads, characterized their cilia driven transport over the airway surface and studied their involvement in clearance of particles from the airways.

## Methods

### Animals

Experimental procedures and protocols were carried out in accordance with the EU Directive 2010/63/EU for the care and use of laboratory animals and the ARRIVE guidelines. Ethical permits were obtained for newborn CFTR null (CF) and wild-type (WT) piglets from Regierungen von Oberbauern, Munich, Germany (AZ55.2-1-54-2531-78-07) and Jordbruksverket, Jönköping, Sweden (Dnr 6.7.18-12708/2019). Protocols for the analysis of explant tissue from weaned pigs (2937/2020) and in vivo bronchoscopy in anesthetized pigs (1763/2018) were approved by the Swedish Laboratory Animal Ethical Committee in Gothenburg, Sweden.

### Preparation of pig airways for microscopy

For experiments on live explants from newborn piglets (*Sus scrofa domesticus*), CF piglets and WT littermates of both sexes were used. CF piglets were generated by breeding male and female heterozygous CF carrier animals from a previously described CF pig model [10]. Intramuscular administration of 0.175 mg Cloprostenol (Estrumate®, Intervet GmbH, Unterschleissheim, Germany), a synthetic analog of prostaglanding F_2α_ on gestation day 112–114 was used to induce birth. For genotyping, tail clippings from the piglets were taken after birth and genomic DNA isolated using Nexttec™ Genomic DNA Isolation Kit (Nexttec GmbH, Leverkusen, Germany) according to the manufacturer’s protocol. Conventional end-point PCR employing primer sets Cg2f/Cg5r for the WT CFTR allele and Cg2f/Cg1r for the knock-out allele was used with primers Cg2f, 5-AGA AGA GTA GGG CCT TTG GCA T-3; Cg1r, 5-TGG CTG AAC TGA GCG AAC AAG T-3; Cg5r, 5-AGC ACA TGT GGG TCT TAG AGT ACG-3. PCR included an initial denaturation of 5 min at 95°, 35 cycles of 15 s at 95 °C, 15 s at 56 °C, 30 s at 72 °C and final steps of 5 min at 72 °C and 5 min at 4 °C. Piglets (CF and WT) were anesthetized by Ketamine (Ursotamin®, Serumwerk Bernburg, Germany) and Azaperone (Stresnil®, Elanco Animal Health, Bad Homburg, Germany) and killed by intracardial injection of Tanax® T61 euthanasia solution (Intervet GmbH, Unterschleissheim, Germany) within 24 h of birth. Airways and lungs were excised and all connective and pulmonary tissue removed under Perfadex® solution, pH 7.2 (XVIVO Perfusion, Gothenburg, Sweden) before the prepared airways including larynx, trachea and bronchi were transferred to a 50 ml tube with Perfadex® solution, pH 7.2 and shipped at 4 °C overnight to Gothenburg.

Female weaned pigs (*Sus scrofa domesticus*) weighing 10–12 kg were acquired from a local farm and allowed to acclimatize for five days. The animals were housed according to Swedish legislation, sedated with an intramuscular injection of 0.6 mg/kg Dexdomitor (Orion Pharma, Danderyd, Sweden) and 0.03 mg/kg Zotelil (Virbac, Kolding, Denmark) and killed by intravenous installation of 200 mg/kg Allfatal (Omnidea, Stockholm, Sweden). Death was ensured by lack of heart sounds and circulatory arrest.

### Live explant video microscopy

#### Low-resolution time-lapse acquisition

The distal trachea and proximal primary bronchi were opened along the smooth muscle to expose the mucosal surface and the live tissue was mounted in a Petri dish coated with Sylgard 184 Silicone Elastomer (Dow Corning, Wiesbaden, Germany) using insect pins (Cat# 26000-25, Agnthos, Lidingö, Sweden). The tissue was kept hydrated by adding a small amount of oxygenated (95% O_2_, 5% CO_2_) Krebs-glucose buffer (116 mM NaCl, 1.3 mM CaCl_2_, 3.6 mM KCl, 1.4 mM KH_2_PO_4_, 23 mM NaHCO_3_, 1.2 mM MgSO_4_, 10 mM d-glucose, 5.7 mM pyruvate, 5.1 mM glutamate, pH 7.4), and gradually heated to 37 ℃. The dish was placed on a table with a 20-degree incline to assure air–liquid interface and mucus transport against gravity.

To visualize the different components of the mucus, tissue was stained with 0.4 mM Alcian blue 8GX pH 7.4 (Cat# A5268, Sigma-Aldrich, St. Louis, MO), 5 µg/ml fluorescein labeled *Lotus tetragonolobus* (LTL) lectin (Cat# FL-1321-2, Vector laboratories, Burlingame, CA) and/or 5 µg/ml DyLight 649 labeled *Ulex europaeus* agglutinin (UEA1) (Cat# DL-1068-1, Vector laboratories, Burlingame, CA) dissolved in oxygenated Krebs-glucose buffer, pH 7.4. Red (580/605) fluorescent beads (Cat. # F8793 FluoroSpheres™ Carboxylate-modified microspheres, 0.04 μm, ThermoFisher Scientific, Waltham, MA) or activated charcoal particles (Cat# 05112-500G, Merck, Darmstadt, Germany) [11] were suspended in oxygenated Krebs-glucose buffer pH 7.4 and added to the explants.

Time-lapse recordings were acquired through an SMZ18 stereo microscope (Nikon, Tokyo, Japan) and white light (Photonics, Pittsfield, MA) or a CoolLED pE-300ultra light source (CoolLED, Andover, UK) using a 5.0-megapixel color CCD camera (DS-Fi2 or DS-Fi3, Nikon, Tokyo, Japan) or a monochrome cooled CCD camera (DS-Qi1, Nikon, Tokyo, Japan) and NIS elements software (RRID:SCR_014329, Nikon, Tokyo, Japan). Mucus bundle and thread thickness was calculated as a mean of at least ten measurement points along the same bundle or thread and presented as one data point. To calculate transport velocity of bundles and threads, the mean of five points along a moving bundle or thread was measured using NIS elements. Either the mean was presented as one data point in order to investigate the variation among different threads, or the mean per pig was calculated for mucus bundles. Movie speed was increased 16× using Microsoft Movie Maker (Microsoft, Redmond, Washington).

#### High-resolution video acquisition

Mid tracheas from weaned pigs, newborn WT and CF piglets were dissected into 1 cm pieces, opened to expose the luminal surface and pinned with insect pins (Cat# 26000-25, Agnthos, Lidingö, Sweden) to Petri dishes coated with Sylgard elastomer (Dow Corning, Wiesbaden, Germany). To visualize the mucus components, fluorescein labeled *Lotus tetragonolobus* (LTL) lectin (Cat# FL-1321-2, Vector laboratories, Burlingame, CA) and DyLight 649 labeled *Ulex europaeus* agglutinin (UEA1) (Cat# DL-1068-1, Vector laboratories, Burlingame, CA) were used alone or in combination and dissolved at 5 µg/ml in Krebs-glucose buffer, pH 7.4 (buffer composition as above). Live explants were incubated for 10 min at ambient temperature with 50 µl lectin solution and 2 ml Krebs-glucose added before imaging.

Videos were recorded via a Zeiss LSM900 Axio Examiner Z1 microscope and Plan-Apochromat × 20/1.0DIC water immersion objective (Cat# 420762-9900-000, working distance 1.8 mm, numerical aperture 1.0, Carl Zeiss, Oberkochen, Germany) using the Zeiss Axiocam 305 color 5-megapixel high-speed camera. This resulted in higher resolution but a smaller field of view and larger magnification than the low-resolution time-lapses recorded with the stereo microscope. Videos were processed with the Zen blue software (RRID:SCR_013672, Carl Zeiss, Oberkochen, Germany) and Adobe Premiere Elements (San José, California). Mucus bundle and thread thickness was calculated by measuring ten points along the mucus formation with ImageJ (https://imagej.nih.gov/ij/index.html) and the mean was used as one data point.

### Live explant image acquisition

Newborn WT piglet tracheas were dissected as for video microscopy into 1 cm pieces, opened and pinned with insect pins to Petri dishes coated with Sylgard. Mucus was stained with fluorescein labeled *Lotus tetragonolobus* (LTL) lectin and/or DyLight 649 labeled *Ulex europaeus* Agglutinin I (UEA1) mixed in oxygenated Krebs-glucose buffer at 5 µg/ml and added to the tissue. Nuclei were stained with Syto 59 at 20 µM (Cat# S11341, ThermoFisher Scientific, Waltham, MA). The tissue was washed after approximately 30 min of incubation at ambient temperature in the dark and 2 ml fresh oxygenated Krebs-glucose buffer was added. Mucus threads were visualized with a Plan-Apochromat × 20/1.0DIC water immersion objective (Cat# 420762-9900-000, Carl Zeiss, Oberkochen, Germany), an upright LSM 700 Axio Examiner 2.1 confocal imaging system (Zeiss LSM 700 microscope, RRID:SCR_017377) or a Zeiss LSM900 Axio Examiner Z1 microscope with the Airyscan 2 imaging system and Zen blue software (RRID:SCR_013672, Carl Zeiss, Oberkochen, Germany). After acquisition, Airyscan images were processed using standard Airyscan processing algorithms.

### Bronchoscopy in anesthetized pigs

Adult female pigs (*Sus scrofa domesticus*) weighing 55–70 kg acquired from a local farm were allowed to acclimatize for five days before experiments, housed according to Swedish legislation and sedated using the same procedure as the weaned pigs. Only female pigs were used due to inconvenience in housing both sexes since they have to be kept separate. To leave the airways free for the bronchoscope, anesthesia was introduced via the intravenous route with 1500 mg/ml Pentobarbital (Apotek Produktion & Laboratorier AB, Kungens Kurva, Sweden) and 250 mg/ml Petidin (Apotek Produktion & Laboratorier AB, Kungens Kurva, Sweden) using a flow of 300 ml/h. During anesthesia, temperature and intra-arterial blood pressure were monitored and the dose adjusted to ensuring lack of reflexes while maintaining spontaneous breathing. Metacam® (Meloxicam, Boehringer Ingelheim Animal Health Nordics A/S, Copenhagen, Denmark) was administered intravenously at 0.4 mg/kg for analgesic purposes. Pigs were placed on their left side, the bronchoscope (PrimeSight, Congentix Medical, Enschede, The Netherlands) positioned with the aid of a laryngoscope and 1 ml charcoal particles suspended in Krebs-glucose buffer were installed via the working channel. The charcoal was washed out of the channel by installing 2 ml physiological saline. Videos were recorded with an endoscopy video processor (DPU-7000, Vision Sciences, Chessington, UK). After bronchoscopy the pigs were killed by intravenous installation of 200 mg/kg Allfatal (Omnidea, Stockholm, Sweden). Death was ensured by lack of heart sounds and circulatory arrest.

### Fluorescent staining and confocal imaging of fixed paraffin sections

Small samples the length of two to three cartilage rings from piglet tracheas were fixed in 4% formalin, embedded in paraffin and cut in 4 μm thick sections. For fluorescent detection, sections were dewaxed using Xylene substitute (Cat# A5597 Sigma-Aldrich, St. Louis, MO) and hydrated in decreasing concentrations of ethanol. Antigen retrieval was performed by microwave heating in 0.01 M citric buffer pH 6.0. for 10 min. Sections were blocked for 60 min with 3% donkey serum in Tris-buffered saline (TBS) and permeabilized with 0.1% Triton X-l00 at ambient temperature. Primary antibodies, mouse monoclonal MUC5AC clone 45M1 (Cat# MA1-38224, RRID:AB_2146842, ThermoFisher Scientific, Waltham, MA) and custom made rabbit anti-human MUC5B directed against the D3 domain were incubated sequentially overnight in blocking solution at 4 °C. The custom-made anti-MUC5B antibody was characterized previously [12]. Donkey anti-rabbit Alexa 488 (Cat# A32790, RRID:AB_2762833) and donkey anti-mouse Alexa 647 (Cat# A-31571, AB_162542, Thermo Fisher Scientific, Waltham, MA) secondary antibodies were incubated in blocking solution for two hours at ambient temperature in the dark to visualize MUC5B and MUC5AC, respectively. Biotinylated *Lotus tetragonolobus* (LTL) lectin (Cat# B-1325-2, Vector Laboratories, Burlingame, CA) or biotinylated *Ulex europaeus* Agglutinin I (UEA1) lectin (Cat# B-1065-2, Vector Laboratories, Burlingame, CA) was incubated in blocking solution for one hour at ambient temperature on dewaxed and rehydrated slides. Streptavidin, Alexa Fluor 488 (Cat# S11223) or 555 (Cat# S21381, Thermo Fisher Scientific, Waltham, MA) conjugate were used to visualize LTL and UEA1, respectively. Nuclei were counterstained with Hoechst 34580 (Cat# 565877, RRID:AB_2869723, BD Biosciences, San Jose, CA) and sections were mounted with Prolong gold mounting medium (Cat# P36934, RRID:SCR_015961, Thermo Fisher Scientific, Waltham, MA). Images were acquired with an upright LSM 700 Axio Examiner 2.1 confocal imaging system, Plan-Apochromat 40x/1.4 Oil DIC M27 (Cat# 420762-9900-000) and Zen blue software (RRID:SCR_013672, Carl Zeiss, Oberkochen, Germany). Images were processed with Imaris software, version 9 (Oxford Instruments, Abingdon, U.K.).

### Electron microscopy

Samples (two to three cartilage rings in length) from newborn piglet tracheas were fixed in modified Karnovsky's fixative (2% paraformaldehyde, 2.5% glutaraldehyde in 0.05 M sodium cacodylate buffer, pH 7.2) for 24 h at 4 °C. Postfixation was performed in 1% OsO_4_ at 4 °C three times with intervening 1% thiocarbohydrazide steps. The samples were dehydrated with increasing concentrations of ethanol followed by hexamethyldisilazane that was allowed to evaporate. Samples were mounted on aluminum specimen pin stubs (Cat# AGG301, Agar Scientific, Stansted, Essex, UK) with carbon tabs (Cat# AGG3347N, Agar Scientific, Stansted, Essex, UK) and conductive silver paint (Cat# 16040-30, Ted Pella, Redding, CA). To decrease charging, samples were sputter-coated with palladium before imaging at 3 kV in a field emission scanning electron microscope (Zeiss DSM 982 Gemini or Zeiss Leo Ultra 55, Carl Zeiss, Oberkochen, Germany).

### Statistical analysis

Statistical analyses were performed using GrapPad Prism version 9 (RRID:SCR_002798, GrapPad, San Diego, CA) and data presented as median with interquartile range. All data points are shown. Each bundle or thread was considered as n = 1 unless otherwise stated. Number of data points and pigs in each group are presented in each figure legend. Differences were assessed with two-tailed non-parametric Mann–Whitney test for comparison of two groups or Kruskal–Wallis and Dunn´s multiple comparisons test for comparisons between multiple groups. The test used is stated in each figure legend. Significance was defined as P ≤ 0.05. No methods of randomization or determination of sample size were used due to limited access to pigs. Because the morphology of newborn CF piglets is unmistakable, the experiments were not performed blinded.

## Results

### Mucus bundles produced by submucosal glands were transported cephalically

**Fig. 1:**
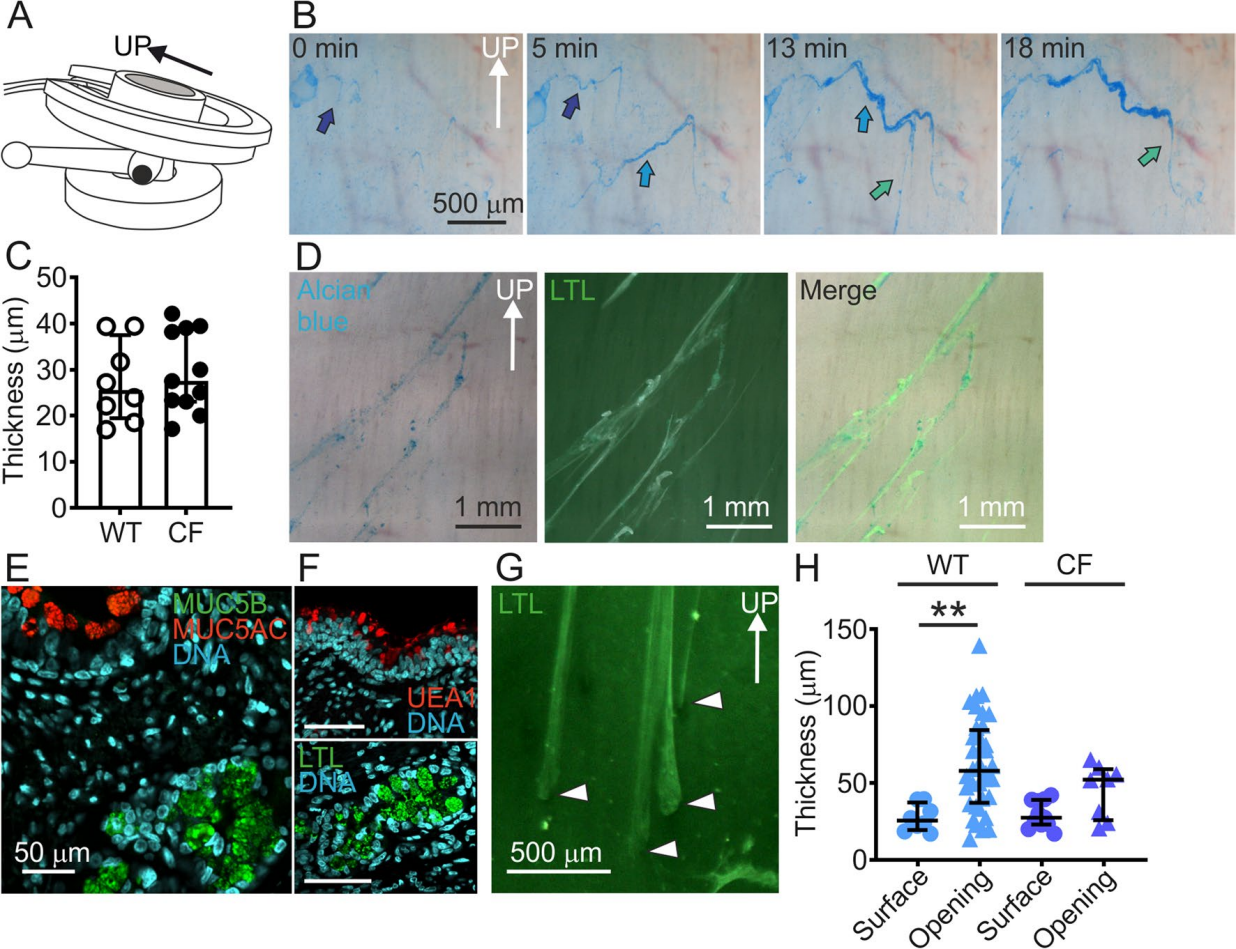
Mucus bundles made by submucosal glands were transported by cilia. **A** Schematic drawing of the tilted table with heating to 37 °C where pig distal trachea and primary bronchi were mounted to ensure air–liquid interface and transport against gravity. **B** Image sequence from two sequential low-resolution time-lapses lasting five minutes each. Alcian blue stained mucus bundles (arrows) on explanted trachea from a weaned WT piglet. Speed of corresponding movies (Additional files 1 and 2) increased 16×. **C** Bundle thickness measured on the airway surface, each data point is the mean of at least ten measurements per bundle, median with interquartile range. WT: 3 piglets, 3 time-lapses, 8 bundles. CF: 3 piglets, 4 time-lapses, 11 bundles. **D** Explanted WT piglet airway mucus bundles stained with Alcian blue (blue), LTL (green) and merged low-resolution image. ** E** Confocal high-resolution image of formalin-fixed paraffin section from a newborn piglet trachea stained with antibodies against MUC5B and MUC5AC, illustrating the production of MUC5B (green) in submucosal mucous cells and mostly MUC5AC (red) in surface goblet cells. Some MUC5B was observed in surface cells. **F** Surface goblet cell mucus stains with the UEA1 lectin (red), whereas mucus in submucosal glands (MUC5B) stains with the lectin LTL (green). **G** Explanted WT piglet airway with LTL stained mucus bundles, low-resolution image. Arrowheads indicate gland openings. **H** Bundle thickness measured on the airway surface compared to at the gland openings. The mean of ten measurements per opening is presented as one data point. Data presented as median with interquartile range. WT: 3 piglets, 9 time-lapses, 36 bundles. CF: 2 piglets, 3 time-lapses, 8 bundles. WT surface vs. WT opening P = 0.0025 **, Kruskal–Wallis and Dunn´s multiple comparisons test

All experiments were performed in porcine distal trachea or primary bronchi. To study mucus transport, explanted distal trachea and proximal primary bronchi were mounted in Sylgard elastomer-coated Petri dishes using insect pins. The tissue was kept hydrated by adding a small volume of oxygenated Krebs-glucose buffer (pH 7.4) containing Alcian blue, heated to 37 ℃ and kept at that temperature throughout the experiments. The mounted tissue was then placed on a stage tilted 20 degrees with the cephalad end of the trachea on the uppermost part of the stage (Fig. 1A). Most of the fluid drained off, to ensure that only a small volume of liquid was retained on the tissue. Time-lapses were recorded via a stereo microscope [3, 7], resulting in low-resolution videos. The Alcian blue stained mucus bundles were transported upward over the airway surface and aggregated into larger mucus formations during the 18 min recording (Fig. 1B, Additional files 1 and 2). We determined the median bundle thicknesses on newborn WT and CF piglet tracheas to be similar, 25.5 µm and 27.5 µm, respectively (Fig. 1C). Simultaneous staining of the explanted trachea with Alcian blue and LTL indicated that both dyes stained the same mucus bundles (Fig. 1D). As demonstrated previously, mucus bundles produced by submucosal glands were based on the MUC5B mucin and stained by the *Lotus tetragonolobus* lectin (LTL) (Fig. 1E) [1], whereas the surface goblet cells produced mostly MUC5AC and minor amounts of MUC5B, both stained by the lectin *Ulex europaeus* agglutinin (UEA1) (Fig. 1F) [3, 7].

To address if the mucus bundles had the same thickness when they emerged from the submucosal gland openings, the bundles were stained with fluorescent lectin (LTL) and visualized at the gland openings (Fig. 1G). To our surprise, the mucus bundles in newborn WT piglet trachea gland openings were twice as thick and more variable in thickness (median 57.9 µm) compared to on the surface (25.5 µm). In contrast to WT, in CF piglets the bundle thickness at the gland-openings was similar to the thickness on the tracheal surface (Fig. 1H).

### Mucus threads were produced by surface goblet cells

**Fig. 2:**
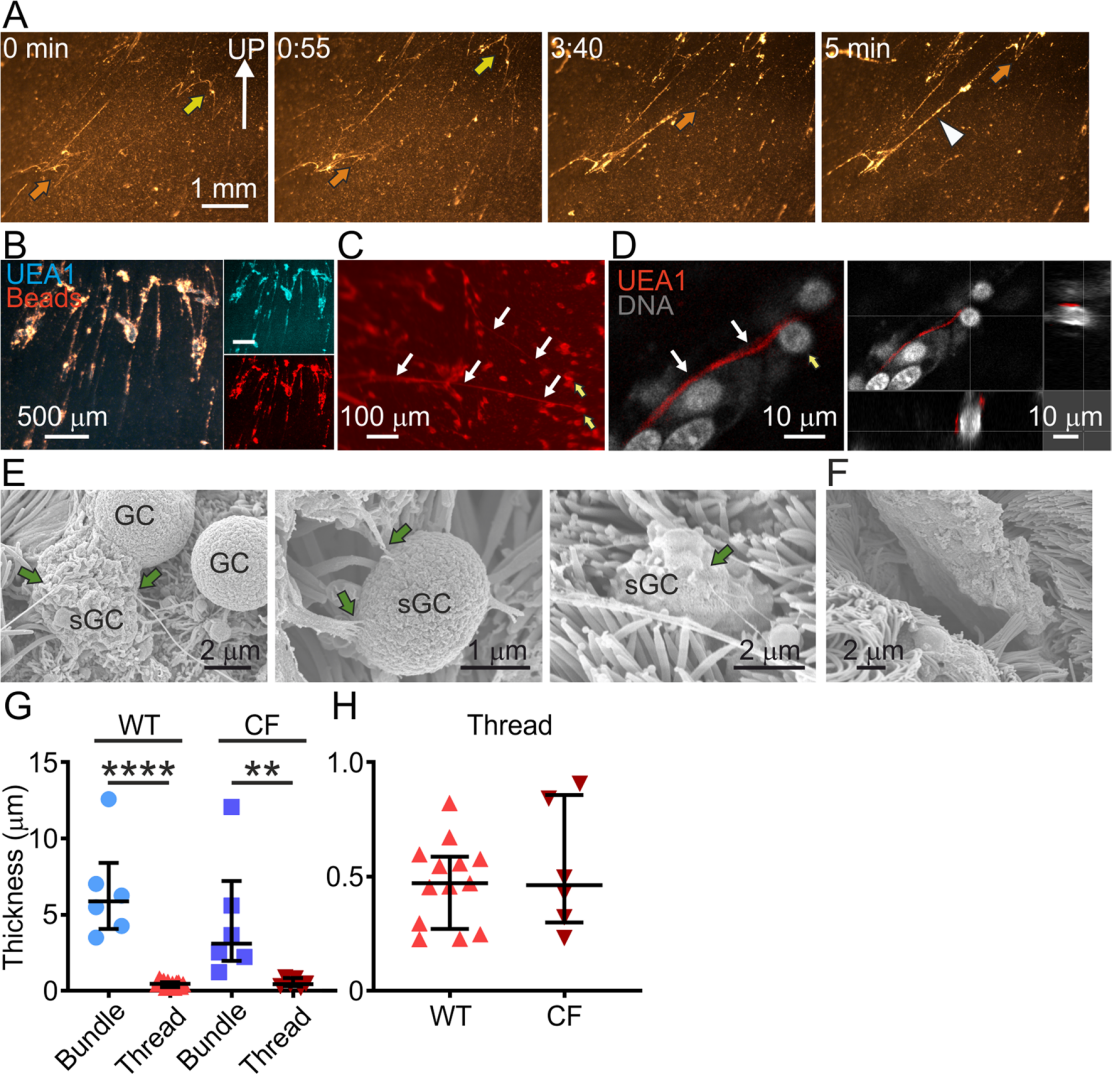
Mucus threads were secreted by surface goblet cells. **A** Image sequence from a low-resolution time-lapse where beads were collected by threads on an explanted WT piglet trachea. Arrow points to moving mucus assemblies and arrowhead to long mucus formation with increasing thickness. Speed of corresponding movie (Additional file 3) increased 16×. **B** The lectin UEA1 (blue) stained bead-collecting assemblies (red), low-resolution image of explanted WT piglet trachea. **C** Low-resolution image of explanted live UEA-stained (red) WT piglet trachea. Yellow arrows indicate surface goblet cells and white arrows point to threads secreted from the goblet cells. **D** High-resolution Airyscan Z-stack of explanted live WT piglet trachea stained with Syto 59 (DNA, grey) and UEA1 (mucus, red). Yellow arrows indicate surface goblet cells and white arrows point to threads secreted from the goblet cells. **E** Scanning electron micrographs from three different WT piglet tracheas. Threads from secreting goblet cells (sGC) are indicated by green arrows. Some goblet cells (GC) were not secreting. **F** Scanning electron micrograph of mucus bundle originating in a submucosal gland. Note that the mucus bundle is thicker than the mucus threads in **E**. Sample from a CF piglet trachea. **G** Comparison of mucus bundle and mucus thread thickness in scanning electron micrographs. One data point represents the mean of 10 measurements per bundle or thread. Mucus threads were thinner than bundles in both WT and CF, Mann–Whitney test: WT bundles vs. WT threads P < 0.0001 ****, CF bundles vs. CF threads P = 0.0022 **. WT bundles: median 5.9 µm, 6 bundles (4 pigs), WT threads: median 0.47 µm, 13 threads (4 pigs), CF bundles: median 3.1 µm, 6 values (4 pigs), CF threads: median 0.46 µm, 6 values (4 pigs). Data presented as median with interquartile range. **H** The thread median thickness was 0.5 µm after fixation

To visualize how the mucus transported beads [3], negatively charged carboxylate-modified fluorescent beads were added to mounted newborn WT piglet trachea before tilting. After tilting, some beads drifted down with gravity, whereas others were transported up against gravity during the five-minute recording (Fig. 2A, Additional file 3). At this low magnification the threads were barely visible, but their location could be deduced by the coordinated movement of beads in rows (yellow arrows). These rows of beads must be collected onto mucus and their thin nature made us call them mucus threads, similar to previous publications [8]. The thicker mucus assemblies (white arrowhead) moved slower (Fig. 2A, Additional file 3). In order to study the origin of the bead-collecting mucus threads in detail with higher resolution and magnification and investigate the relationship between surface goblet cell mucus and the bead-gathering threads, the UEA1 lectin was added together with the fluorescent beads. We have repeatedly demonstrated that this lectin stained mucins produced by piglet airway surface goblet cells [7], (Fig. 1F). The same threads gathered beads and stained with UEA1, as illustrated in Fig. 2B. Low-resolution microscopy (Fig. 2C) and high-resolution Airyscan images (Fig. 2D) of live piglet airways revealed that the mucus threads (white arrows) originated in surface goblet cells (yellow arrows). The threads (green arrows) could also be observed in scanning electron micrographs (Fig. 2E) emerging from secreting goblet cells (sGC) in fixed tissue from newborn piglet tracheas. The threads were clearly thinner than the mucus bundles observed in the same type of preparation (Fig. 2F). To illustrate this further, the thickness of mucus threads and bundles was quantified in scanning electron micrographs from newborn WT and CF piglet tracheas (Fig. 2G). Each thread from surface goblet cells or mucus bundle emerging from submucosal glands was measured in ten places and the mean presented in Fig. 2G. Threads were thinner than mucus bundles in both WT and CF and as the mucus threads were very thin in comparison to the bundles. The mucus thread thickness is presented with a different scale on the y-axis in Fig. 2H to visualizes the variability in thickness. In these fixed samples, the median mucus thread thickness was 0.5 µm and the median mucus bundle thickness was 5.9 µm. Using these results, we calculated a factor for the fixation induced shrinkage to 4 for fixed mucus bundles (5.9 µm) compared to live explants stained with Alcian blue, 25.5 µm (Fig. 1H). Using this shrinkage factor, we estimated the theoretical thickness of hydrated mucus threads to 2 µm (4 × 0.5 µm). We found it important to have such a guideline to define mucus threads as multiple threads quickly gathered into mucus assemblies. We thus defined mucus threads as linear structures with a maximum of 2 µm diameter in live tissue. Please observe that all mucus threads in Fig. 2H were below 1 µm thick and thus conformed to the definition mucus threads.

### Mucus threads were closer to the epithelium and thinner than mucus bundles

**Fig. 3:**
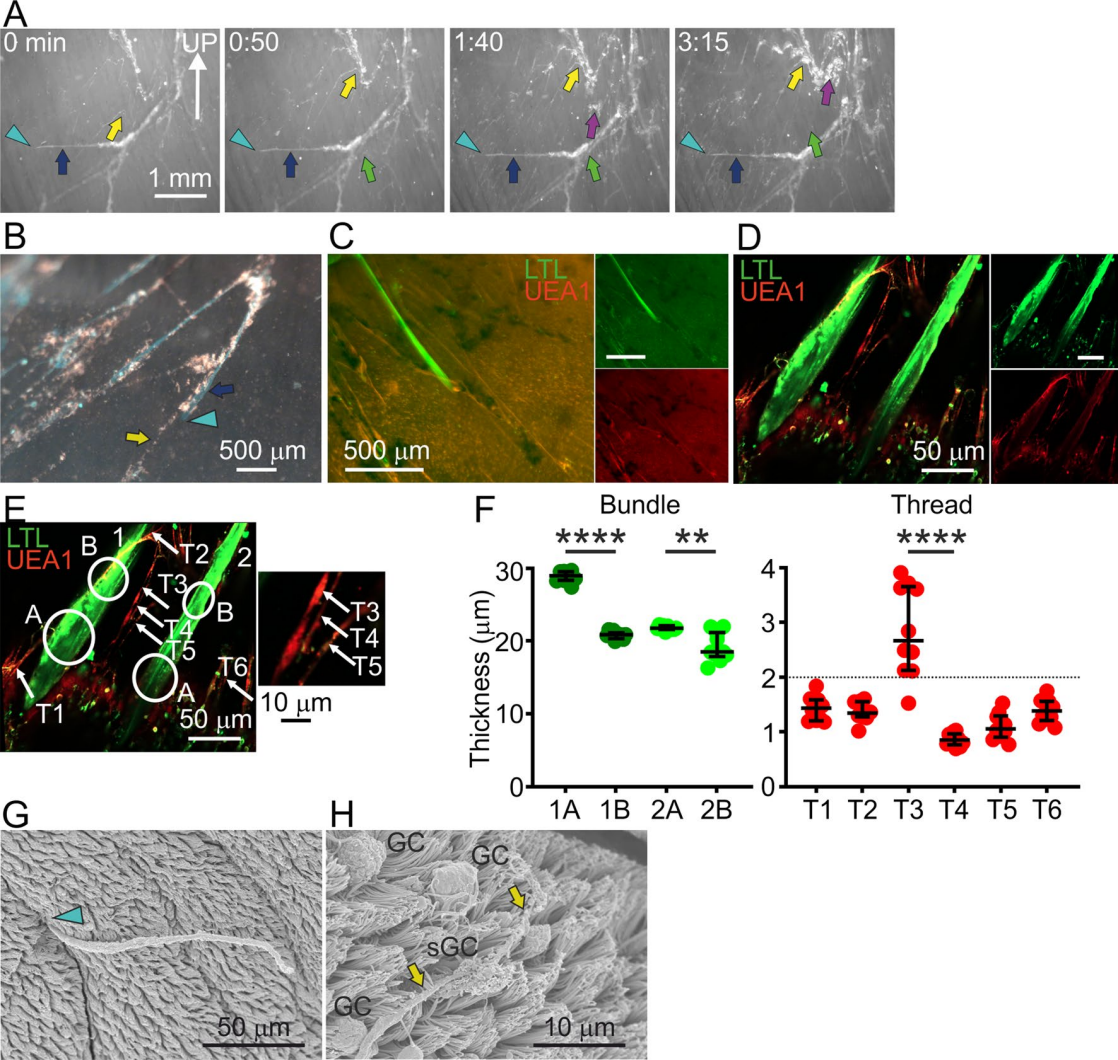
Mucus threads from surface goblet cells were thinner than mucus bundles. **A** Image sequence from a low-resolution time-lapse where threads collected beads on an explanted WT piglet trachea. Speed of corresponding movie (Additional file 4) increased 16×. Mucus bundle coming out of a gland opening indicated by light blue arrowhead. Dark blue arrow: immobile bundle. Yellow, green and purple arrows indicate different moving mucus assemblies. **B** Low resolution image of bundles (LTL, blue arrow) and beads gathered into threads (yellow arrow) on WT piglet tracheas (arrowhead: gland opening). **C** Low-resolution image of explanted WT piglet trachea with bundle from a submucosal gland, LTL (green) and threads, UEA1 (red). **D** Airyscan high-resolution Z-stack of LTL-stained bundles (green) from submucosal glands and UEA1-stained threads (red) in a live explanted WT piglet trachea. **E** Thickness analysis of mucus bundles (LTL, green) and mucus threads (UEA1, red) in the image in **D**. Inset: zoom of the threads in the image center. **F** Mucus bundle thickness was calculated as a mean of 10 measurements close to the gland opening (1A, 2A) and further along the bundle (1B, 2B), as indicated in (**E**). Bundles were thicker close to the gland opening, Mann–Whitney test: 1A vs. 1B P < 0.0001 ****, 2A vs. 2B P = 0.0052 **. Thread thickness was calculated in the same way, as indicated in (**E**). Based on bundle thickness in live explants compared to fixed scanning electron micrographs, the maximum mucus thread thickness was determined to 2 µm (dashed line). Thread T3 was thicker than T4, Mann–Whitney test P < 0.0001 ****. Bundles were thicker than threads, Mann–Whitney test P = 0.0095 **. Data presented as median with interquartile range. **G** Scanning electron micrograph from a WT piglet trachea with mucus bundle from a submucosal gland (blue arrowhead). **H** Scanning electron micrograph of WT piglet trachea mucus thread (yellow arrows), GC: goblet cell, sGC: secreting goblet cell

The mucus bundles were observed further away from the epithelial surface in a higher focal plane than the mucus threads, whereas the mucus threads were found to move closer to the epithelial surface, as exemplified by an image sequence from a low-resolution time-lapse recording made on a newborn WT piglet trachea (Fig. 3A, Additional file 4). The mucus threads visualized by gathered fluorescent beads (yellow, green and purple arrows, Fig. 3A) moved underneath the mucus bundle (blue arrow). The definition as mucus bundle was confirmed by its visible connection to a gland opening (blue arrowhead). In live newborn WT piglet explants (Fig. 3B), beads gathered on thinner threads (yellow arrow) whereas the LTL lectin stained thicker mucus bundles (blue arrow) originating in submucosal glands (blue arrowhead). With time, the threads were trapped and collected on the mucus bundles (Fig. 3B), as can be observed in Additional file 4, Fig. 3A. The fluorescent beads did not bind directly to the mucus bundles, as demonstrated previously [3, 7]. Using low-resolution imaging, UEA1-positive mucus threads were visualized on the surface of a newborn WT piglet trachea where the mucus bundles were stained with the LTL lectin (Fig. 3C). The UEA1 stained mucus threads and LTL positive mucus bundles were also observed using the high-resolution Airyscan technique (Fig. 3D). Thus, mucus threads were thinner than mucus bundles in both fixed tissue as observed by scanning electron microscopy (Fig. 2F and G) and on hydrated live tissue as observed by different types of light microscopy (Fig. 3A–D).

To further highlight the difference in thickness between bundles and threads, we measured the thickness of the two bundles visible in Fig. 3D in two positions, close to the gland opening and on the surface, as indicated in Fig. 3E and plotted in Fig. 3F. Similarly, the thickness of six threads was calculated and plotted in Fig. 3G. The only thread above our arbitrary 2 µm cutoff for threads in this image was T3. The inset of Fig. 3E emphasizes that this thread had collected additional material, further strengthening our model of mucus threads being below 2 µm thick and mucus collections thicker than 2 µm being mucus assemblies. Comparing mucus bundles (Fig. 3G) and mucus threads (Fig. 3H), the difference in thickness and origin was obvious in scanning electron micrographs of a mucus bundle emerging from a submucosal gland (blue arrowhead) and mucus threads (yellow arrows) emerging from a secreting surface goblet cell (sGC). In this case, the mucus threads had again accumulated into thicker mucus assemblies (yellow arrows).

### Thicker mucus assemblies docked onto mucus bundles

**Fig. 4:**
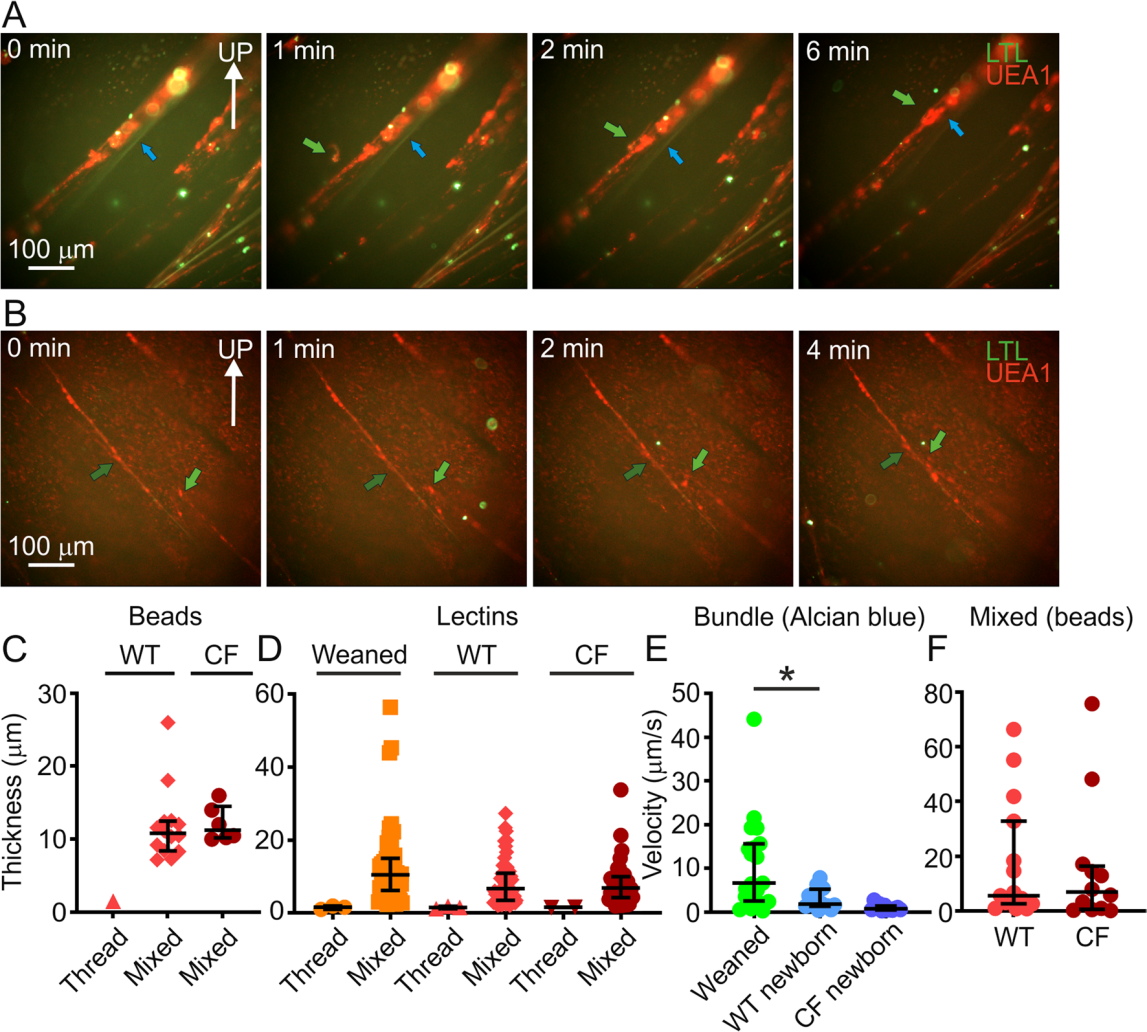
Mucus threads collected together and on mucus bundles. **A** Image sequence from a high-resolution video illustrating a UEA1-positive mucus assembly (green arrow) docking to an LTL-positive bundle (blue arrow) coated with UEA1 on a newborn WT piglet trachea (Additional file 5). **B** Image sequence from a high-resolution video illustrating a UEA1-positive mucus assembly (green arrow) docking to another UEA1-positive mucus assembly (dark green arrow) on a newborn WT piglet trachea (Additional file 6). **C** Bead-gathering mucus formations were divided into threads and mucus assemblies based on the maximum thread thickness of 2 µm determined by the difference in thickness between hydrated and fixed mucus. One thread was observed in WT and none in CF. WT: 15 values (9 pigs), CF: 6 values (3 pigs). **D** Thickness measurements of UEA1-stained mucus in high-resolution images from live explant tracheas from weaned pigs, newborn WT and CF piglets resulted in 3 threads and 48 mucus assemblies (6 weaned pigs), 3 threads and 54 mucus assemblies (10 newborn WT piglets), 2 threads and 37 mucus assemblies (12 newborn CF piglets), using the same criteria as in C. Mucus assemblies in weaned pigs were thicker than the corresponding assemblies in newborn CF piglets, P = 0.0418 * Kruskal–Wallis and Dunn´s multiple comparisons test. **E** Alcian blue stained mucus bundle velocity in weaned, newborn WT and CF tracheas measured in low-resolution time-lapses recorded on the tilted table. Each data point represents one pig. Weaned 19 pigs, newborn WT 14 pigs and newborn CF 12 pigs. Weaned bundles were faster than newborn WT bundles, P = 0.0111 *, Mann–Whitney test. **F** There was no difference in velocity between bead-collecting mucus assemblies in newborn WT and CF piglet trachea. WT: 15 values, 15 time-lapses, 8 pigs. CF: 12 values, 12 time-lapses, 6 pigs. Mann–Whitney test: P > 0.05. Data in (**C**–**F**) presented as median with interquartile range

Using higher magnification, mucus assemblies were observed docking to mucus bundles, as illustrated in the image sequence from a newborn WT piglet trachea (Fig. 4A). The mucus bundle with a median diameter of 25 µm was stained with LTL (blue arrow) and the mucus assembly with a median diameter of 5 µm was stained with UEA1 (green arrow, Fig. 4A, Additional file 5). It was defined as a mucus assembly because the thickness was above 2 µm. The mucus bundle in this image sequence had already collected UEA1-stained material before the movie was recorded (Fig. 4A). This confirms our previous finding that mucus bundles consisted of a core of MUC5B from submucosal glands and a coating of mucus from surface goblet cells [7].

The mucus threads gathered not only on mucus bundles but also together into larger formations consisting of assemblies of mucus threads, as illustrated by a high-resolution image sequence where a UEA1-stained mucus assembly (green arrow, median thickness 3 µm) docked to another UEA1-stained mucus assembly (dark green arrow, median thickness 5 µm, Fig. 4B, Additional file 6).

As indicated above, the threads visualized by beads were barely visible at low resolution and calculating their thickness resulted in mainly mucus assemblies (Fig. 4C). Only one mucus thread with a thickness below 2 µm was observed in newborn WT piglets and no mucus threads were detected in newborn CF piglets. At higher magnification, lectin staining resulted in detection of only three threads in weaned pigs and newborn WT piglets, respectively and two threads in newborn CF piglets. The rest were mucus assemblies as their thickness was above 2 µm (Fig. 4D). Mucus bundle velocity, quantified on live explants stained with Alcian blue and imaged using low-resolution stereo microscopy was previously reported to be 0.34 ± 0.1 mm/min (mean ± SEM) in WT piglets [7] and slower in CF [3]. Quantifying mucus bundle transport velocity in weaned pigs as well as newborn WT and CF piglets (Fig. 4E) resulted in a median bundle transport velocity of 6.7 µm/s in weaned pigs, 1.9 µm/s in WT and 0.81 µm/s in CF piglets. The bundle transport velocity was clearly higher in weaned pigs, indicating that mucus bundle velocity may change with age and increase as the piglets grow. The mucus bundle velocity was higher in newborn WT piglets compared to CF piglets, but the mucus assembly velocity was similar (Fig. 4F), median 5.6 µm/s for WT and 6.9 µm/s for CF.

### Particles were collected by mucus assemblies

**Fig. 5:**
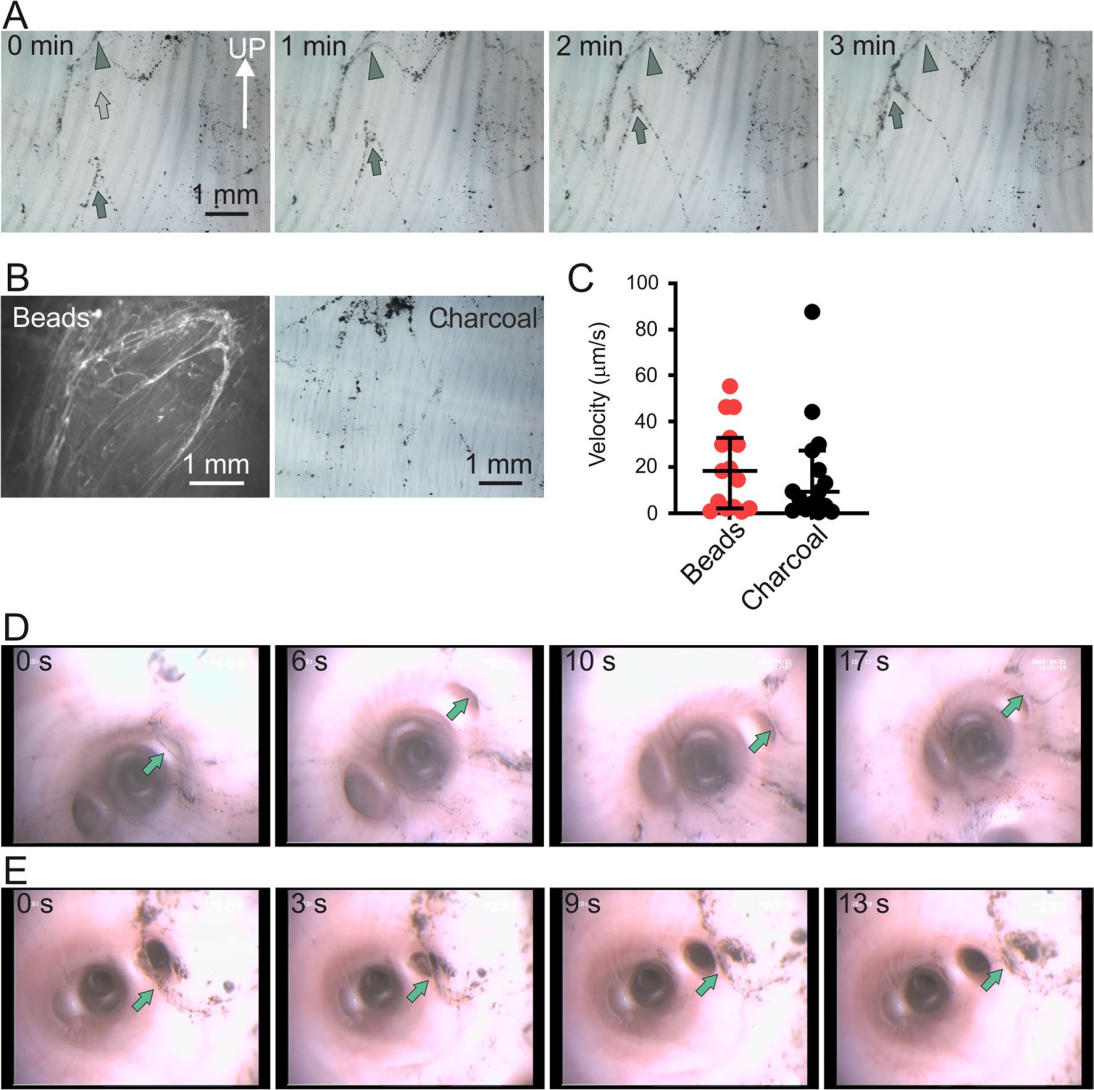
Mucus assemblies collected charcoal particles and fluorescent beads. **A** Image sequence from a low-resolution time-lapse recording to illustrate mucus assemblies collecting charcoal particles added onto explanted trachea from a newborn WT piglet. Grey arrow points to fast-moving mucus assemblies and arrowhead indicates slow-moving mucus assemblies. Speed of corresponding movie (Additional file 7) increased 16×. **B** Mucus clearance pattern visualized using beads or charcoal. **C** Graph comparing transport velocity of mucus assemblies collecting beads (15 values, 9 pigs) or charcoal (15 values, 7 pigs). Collected beads or charcoal moved with the same velocity, Mann–Whitney test P > 0.05. **D** Image sequence from movie (Additional file 8) recorded in vivo in an anesthetized pig (56 kg). **E** Image sequence from movie (Additional file 9) recorded in vivo in an anesthetized pig n. Light green arrows point to moving mucus with collected threads and/or bundles in (**D** and **E)**

Since the fluorescent beads were quickly collected by mucus threads and mucus assemblies, we asked whether charcoal particles would be collected and transported in a similar manner. To study particle clearance, we added charcoal particles of the same type used to study intestinal mucus [11] to live newborn WT piglet tracheal explants and followed the transport by low-resolution time-lapse recording (Fig. 5A, Additional file 7). At this magnification, the charcoal particles were observed to rapidly collect into coordinated rows moving cephalically with the flow generated by the cilia, indirectly visualizing mucus threads. As observed with the fluorescent beads, these rows of charcoal gathered into fast-moving mucus assemblies (grey arrows) which then gathered into larger, slower-moving mucus formations (grey arrowhead), as observed for the fluorescent beads (Fig. 3A). Comparing the clearance pattern of beads and charcoal (Fig. 5B), we concluded that both beads and charcoal particles added to the tracheal surface were quickly collected by the mucus threads, which gathered into mucus assemblies and then onto larger mucus formations. Thus, two different kinds of particles were cleared in a similar pattern. The clearance pattern also illustrated that the mucus did not appear as a layer, as has previously been claimed. The velocity of mucus assemblies visualized by beads and charcoal were quantified (Fig. 5C) and no difference in transport velocity between bead-collecting and charcoal-collecting assemblies was detected.

### Airway mucus clearance in vivo

As mucus clearance in explant tissue may be different from transport in vivo, we wanted to demonstrate particle clearance in live, anesthetized pigs using bronchoscopy. We performed camera-guided bronchoscopy on live anesthetized juvenile pigs and installed activated charcoal via the working channel of the bronchoscope. The charcoal particles were quickly organized on the airway surface into fast-moving rows with apparently coordinated movement, indicating that they gathered on mucus threads. As on the explants, we could observe how the charcoal gathered first into thinner structures (Fig. 5D, 0 s) and then into larger complexes which were transported toward the larynx (Fig. 5D, 17 s). Both image sequences, from two different pigs on two consecutive days, illustrate the collection and transport of charcoal particles to clear the airways in vivo (Fig. 5D, Additional file 8 and Fig. 5E, Additional file 9). The magnification and resolution of the videos recorded via the bronchoscope are not high enough to visualize mucus threads, but the pattern of charcoal movement was similar on the explants and in vivo. We conclude that airway mucus consists of mucus bundles from submucosal glands and mucus threads from the surface goblet cells. These accumulate into larger mucus formations. These observations indicate that mucus does not form a layer in normal airways.

## Discussion

The mucociliary system consists of the cilia surrounded by the periciliary liquid (PCL), the surface liquid and the mucus. The cilia are coated with transmembrane mucins to prevent inhaled particles from penetrating to the epithelial cells and also create a low friction environment to allow ciliary beating [13]. The ciliary beating propels the surface liquid and mucus cephalically to clear the airways. These facts are generally accepted. However, the nature and organization of the airway mucus in the surface liquid is less thoroughly described and often misunderstood. Exposure to inhaled particles and microorganisms starts at birth and with age may induce changes in mucus organization, most dramatically observed in muco-obstructive airway diseases such as CF and COPD [14]. We and others [3, 9] have been forced to use newborn CF piglets to study CF airways as the CF piglets die within a day after birth due to intestinal problems. This has been fortunate, as we have been able to investigate and begin to understand how the non-challenged respiratory tract works before it has been affected by the constant contact with inhaled microbes, pollutants and particles.

The secreted polymeric gel-forming mucins MUC5B and MUC5AC are the main mucins in airway mucus [15]. These mucins consist of similar types of subunits, but their morphology and polymeric network arrangement differ [8]. The cartilagous airways in humans and pigs have a large number of submucosal glands [16], approximately one gland opening per mm^2^ of airway surface [17]. Mucous cells in submucosal glands produce MUC5B, which polymerizes in the N- and C- termini to form long linear polymers. The mucus cells are situated closer to the gland opening than the CFTR-expressing serous cells secreting bicarbonate, chloride and water into the gland duct. The flow of bicarbonate-rich fluid pulls out the linear MUC5B polymers and in the gland duct mucus bundles are formed by parallel molecules of MUC5B [1]. These bundles then sweep with uneven speed across the airways [7].

To study the dynamic movement of mucus bundles, we have mounted live explanted distal tracheas with primary bronchi in a dish and places in a heating block on a 20-degree incline with the cephalad part of the trachea on the highest end. The tilt ensured that superfluous liquid drained off to resemble air–liquid interface [3, 7]. Staining with the positively charged dye Alcian blue or the fluorescent lectin LTL [3, 7] resulted in visualization of mucus bundles emerging from the submucosal gland openings. We have previously demonstrated that mucus bundle transport was faster in live explants from newborn WT tracheas compared to newborn CF tracheas [3], a result consistent with data recorded in the same type of tissue preparation using micro-optical coherence tomography (µOCT) [18]. Now we show that transport of mucus bundles was faster in weaned pig tracheas compared to newborn WT tracheas. Further studies are required to validate and explain this observation as there are indications that mucociliary clearance decreases in the elderly [19, 20], but we could not find any information on mucus transport in newborn children compared to toddlers, older children or adults.

We quantified the mucus bundles to 58 µm in diameter at the gland openings. Underway on the airway surface, the bundles seemed to be pulled out because they became thinner, approximately 26 µm in diameter when measured on the airway surface. The thinning of the mucus bundles was only observed in WT, where the mucus bundles moved and not in CF, where they were immobile [3]. We speculate that lack of CFTR in the serous cells of the submucosal glands and the resulting low levels of HCO_3_^−^, Cl^−^ and water could limit the expansion of the mucin at secretion and generate a more condensed mucus bundle, as supported by studies of CF intestine and lung [21, 22]. The CF mucus bundles may also yield less to the pulling forces of the cilia. Pulling out the WT mucus bundles and making them thinner may result in different properties compared to when the bundles exited the glands. Once on the tracheobronchial surface, these bundles sweep over the airway surface [9] to collect bacteria [3] and larger particles, but as the CF bundles were not pulled out, their properties may be different compared to WT.

Thin mucus threads secreted from surface goblet cells have been suggested to be part of the airway mucus [8]. These threads may consist of MUC5AC [8], MUC5B [12], or both [8, 12] as they emerged from surface goblet cells [8] and in mice deficient in Muc5b, short threads were observed, whereas in mice lacking Muc5ac, the threads were longer and connected to the surface goblet cells [12]. Now we demonstrated that fluorescent carboxylate-modified beads were gathered by the mucus threads secreted from surface goblet cells. Observing mucus threads emerging from surface goblet cells in live explants was possible because the threads could be stained with the fluorescent lectin UEA1 [3, 7] in tracheas from weaned pigs, newborn WT and CF piglets and visualized with the high-resolution Airyscan technique.

To visualize moving mucus threads in explants we used two main techniques, low-resolution time-lapse recordings of fluorescent carboxylate-modified beads and charcoal in explants on a tilted table, with a magnification of 20× and lectin staining in explants imaged in high-resolution using a 20× water immersion objective, resulting in 200× magnification. Unfortunately, it was not possible to retain the highest Airyscan resolution while recording the fast movement of the mucus threads, making it impossible to accurately calculate the thickness and velocity of moving mucus threads.

To address the problem of classifying the observed mucus into mucus threads or mucus assemblies, we used scanning electron micrographs to quantify bundle and thread thickness and calculated a shrinkage factor based on bundle thickness in the fixed samples compared to Alcian blue stained bundles in live, hydrated explants. The shrinkage factor was then used to calculate a maximum thickness for hydrated mucus threads, 2 µm. Mucus formations above this thickness were considered mucus assemblies. This distinction seems reasonable, as calculating mucus thread thickness in a high-resolution Airyscan image where threads could be resolved, resulted in the identification of one mucus assembly where the thickness was calculated to above 2 µm. In scanning electron micrographs, we also demonstrated that the threads were thinner than the mucus bundles in newborn WT and CF piglet tracheas, in addition to having their origin in different anatomical structures.

Measuring mucus thread thickness in live explants proved to be difficult. First, we tried to measure the thickness of bead-gathering formations, but could identify only one thread. The remaining formations were likely mucus assemblies as their thickness was above 2 µm. Then, we tried to measure thread thickness in UEA1 stained weaned pigs, WT and CF piglets and found 8 threads compared to 139 mucus assemblies. These results emphasize our conclusion that mucus threads quickly gather into mucus assemblies and larger formations.

We and others have observed that during transport, mucus bundles became coated by MUC5AC from surface goblet cells [3, 7, 8] and here we demonstrated both that mucus threads gathered into mucus assemblies and that mucus assemblies gathered onto bundles. Thus, we suggest that part of the mucus bundle coating originates in mucus threads. However, since the surface goblet cells make both MUC5AC and MUC5B, lectins could not distinguish between MUC5AC and MUC5B as the mucin glycosylation will be the same when made by the same cell. Specific probes to visualize MUC5AC and MUC5B in live tracheas are needed to elucidate the mucin composition of the threads, mucus assemblies as well as the larger mucus formations. Still, we observed that the threads gathered material when transported over the airway surface, as the thickness of two randomly chosen mucus assemblies was doubled over 5 min.

Even with high-resolution video recording of live explants, the identity, thickness or velocity of mucus threads or bundles could not be determined with sufficient accuracy, as mucus bundles and threads gathered into larger mucus formations over time. However, we could still visualize an important function of the mucus threads, to quickly collect and clear fluorescent beads and charcoal particles introduced to the surface of explanted tracheas. Interestingly, in contrast to bundles, mucus threads moved with similar velocity in newborn WT and CF piglet tracheas. This may be one of the reasons for the historical difficulties in establishing decreased mucociliary clearance as an original defect in CF [23, 24].

We were of course interested in studying the mucus movement in vivo, prompting us to use bronchoscopy in spontaneously breathing pigs on intravenous anesthesia. Charcoal particles were installed via the working channel and could be observed to collect onto moving mucus assemblies resembling what was observed in explants. The resolution of the camera was not high enough to resolve the individual mucus threads, but charcoal particles could be observed moving together in rows as if connected, in conformity with what was observed in live explants. These results confirm the importance of the mucus assemblies for clearance of inhaled particles and validates the observations made in live explants.

## Conclusions

Mucus threads had their origin in airway surface goblet cells as opposed to mucus bundles from submucosal glands. Mucus threads gathered into larger mucus assemblies and coated mucus bundles. We speculate that this organization facilitates mucociliary clearance.

## Supplementary Information

**Additional file 1:** Alcian blue stained mucus bundle transport on an explanted weaned pig trachea, duration 0–5 min, 16× normal speed. Extracted images in Fig. 1B.

**Additional file 2:** Alcian blue stained mucus bundle transport on the same explanted weaned pig trachea as in video 1, duration 13–18 min, 16× normal speed. Extracted images in Fig. 1B.

**Additional file 3:** Mucus assemblies collecting fluorescent beads on an explanted WT piglet trachea, duration 5 min, 16 × normal speed. Extracted images in Fig. 2A.

**Additional file 4:** Mucus assemblies with collected fluorescent beads on an explanted WT piglet trachea, duration 5 min, 16 × normal speed. Extracted images in Fig. 3A.

**Additional file 5:** Mucus assemblies (UEA1, red) attach to a mucus bundle (LTL, green) on an explanted WT piglet trachea. Movie acquired with a 20 × water immersion objective. Extracted images in Fig. 4A.

**Additional file 6:** Mucus assemblies (UEA1, red) attach to a long mucus thread (UEA1, red) on an explanted WT piglet trachea. Movie acquired with a 20 × water immersion objective. Extracted images in Fig. 4B.

**Additional file 7:** Mucus assemblies collecting charcoal particles on an explanted WT piglet trachea, duration 5 min, 16 × normal speed. Extracted images in Fig. 5A.

**Additional file 8:** Mucus assemblies collecting charcoal particles in an anesthetized pig (56 kg). Extracted images in Fig. 5D.

**Additional file 9:** Mucus assemblies collecting charcoal particles in an anesthetized pig (65 kg). Extracted images in Fig. 5E.

## Data Availability

The datasets used and analyzed during the current study are available from the corresponding author on reasonable request.

## Abbreviations

ASL: Airway surface liquid
CF: Cystic fibrosis
CF piglet: Cystic fibrosis transmembrane conductance regulator null piglet
CFTR: Cystic fibrosis transmembrane conductance regulator
GC: Goblet cell
LTL: *Lotus tetragonolobus* Lectin
µOCD: Micro-optical coherence tomography
MUC5AC: Mucin 5 Subtype AC
MUC5B: Mucin 5 Subtype B
PCL: Periciliary liquid
sGC: Secreting goblet cell
UEA1: *Ulex europaeus* Agglutinin
WT: Wild-type
